# Role of lactoferrin in periodontal disease: A meta-analytical assessment of its reliability as a biomarker

**DOI:** 10.3892/mi.2026.307

**Published:** 2026-03-04

**Authors:** Gauri Vilas Patil, Jaideep Mahendra, Deepa Ponnaiyan, Rajan Krishna Kumar, Sakthivel Kannan, Aarudra Devi Jeyaganesh, Kaustubh Suresh Thakare, Bawatharani Maharavi, Aisvarya Naagendran

**Affiliations:** 1 Department of Periodontics, Meenakshi Ammal Dental College and Hospital, Meenakshi Academy of Higher Education and Research, Chennai, Tamil Nadu 600069, India; 2 Department of Periodontics, Sri Ramaswamy Memorial Dental College Ramapuram, Chennai, Tamil Nadu 600089, India; 3 Department of Periodontics, Vidarbha Youth Welfare Society's Dental College and Hospital, Amravati, Maharashtra 444602, India

**Keywords:** periodontitis, lactoferrin, biomarker, saliva, gingival crevicular fluid, serum

## Abstract

The present systematic review and meta-analysis assessed the clinical utility of lactoferrin as a biomarker for periodontitis by evaluating its association with established periodontal parameters, including probing pocket depth (PPD), clinical attachment loss (CAL) and gingival index (GI). The PICOS question was: ‘Are lactoferrin levels different between individuals with periodontitis and periodontally healthy controls, and are these levels associated with disease severity and established clinical periodontal indicators, supporting their role as diagnostic or prognostic biomarkers for periodontitis?’. The present meta-analysis adhered to the Preferred Reporting Items for Systematic Reviews and Meta-Analyses guidelines and was formally registered in PROSPERO (CRD420251003457). A total of 384 articles were initially identified through various databases, from which 15 studies were selected. A random-effects model was applied to analyse lactoferrin concentration in saliva, serum and gingival crevicular fluid in relation to periodontitis and its periodontal parameters, using standardized mean differences (SMDs) with 95% confidence intervals (CIs). Heterogeneity and potential publication bias were examined through statistical methods, including funnel plots, forest plots, Egger's regression analysis, and Begg's rank correlation test. From the initial pool of 384 studies, 15 satisfied the inclusion criteria for the present meta-analysis, comprising a total of 798 participants (496 patients with periodontitis and 302 healthy controls). The combined SMD was 2.630 (P=<0.010; 95% CI: 1.140-11.180), demonstrating a significant link between elevated lactoferrin levels and periodontitis compared with the controls. Egger's regression test produced a t-statistic of -0.646 (P=0.529), reflecting a lack of significant evidence for publication bias. In addition, there was a significant association between lactoferrin concentration and periodontal indicators such as PPD, CAL and GI. In conclusion, lactoferrin showed potential as a biomarker for periodontal disease, with elevated levels consistently linked to its presence and disease severity. However, significant variability in reported levels across studies and a lack of methodological standardization currently limit its diagnostic reliability.

## Introduction

Periodontitis is a disease characterized by inflammation with multiple aetiological factors, resulting in the deterioration of tissues encasing the tooth structure, including the gingiva, alveolar bone, cementum and periodontal ligament. According to estimates from the Global Burden of Disease 2019 study, periodontal disorders influence the health of >1 billion individuals worldwide (1). The inflammatory response of the host immune system to bacteraemia, although protective, can promote chronic infection by modulating biofilm formation and bacterial virulence, thereby disrupting crucial homeostatic mechanisms essential for maintaining periodontal health (2). As the disease progresses, inflammatory and resident cells in the periodontium secrete or activate mediators such as prostaglandin E2, cytokines, chemokines, matrix metalloproteinases and signalling proteins, working together to accelerate the destruction of both soft and hard tissues (3,4). Early identification of inflammatory diseases and prompt treatment can markedly improve health outcomes and enhance disease prognosis.

A biomarker is a characteristic that is objectively measured and evaluated as an indicator of normal biological processes, pathogenic processes or pharmacological responses to a therapeutic intervention (4). Biomarkers can be derived from multiple sources, such as saliva, serum, gingival crevicular fluid (GCF) and dental plaque (3). They are employed for prompt identification of disease, monitoring its progression, predicting potential outcomes, evaluating treatment efficacy and customizing medical interventions (4). During infection, the immune system initiates an inflammatory response by activating various immune cells, including lymphocytes, plasma cells and macrophages, and releasing cytokines. This immune activation leads to the release of multiple inflammatory biomarkers into the bloodstream. Maintaining equilibrium between pro-inflammatory and anti-inflammatory markers is crucial for modulating the host immune response to antigenic challenges, ensuring an effective yet controlled defence mechanism (5).

Lactoferrin is a glycoprotein from the transferrin family that has a high capacity to bind iron. It is produced by exocrine glands and discharged by immune cells such as neutrophils, particularly at sites of infection or inflammation (6). Lactoferrin is found in various body fluids including saliva, dental plaque, GCF, digestive secretions, bronchial secretions, bile, urine, breast milk, serum and tears (7). The primary function initially attributed to lactoferrin was its antimicrobial activity, driven by its capacity to bind iron, which is crucial for the multiplication and survival of bacteria. It also has a key function in numerous bodily processes, including the uptake of iron in the intestines, immune system modulation, anti-viral and anti-oxidant activities (8). Lactoferrin concentrations have been reported to be increased in stimulated whole saliva of individuals with chronic periodontitis and are positively associated with a probing pocket depth (PPD) of ≥6 mm (9). In addition, a previous study demonstrated that lactoferrin levels are elevated in patients with periodontitis relative to healthy individuals, whereas they decline after periodontal therapy (10). Furthermore, higher lactoferrin levels are associated with worse clinical parameters both pre- and post-therapy. Ramenzoni *et al* (11) reported elevated lactoferrin levels in the periodontal pockets of patients with periodontitis, with increased concentrations also observed at clinically healthy sites within the same subjects. These findings suggest that the role of lactoferrin in periodontitis remains unclear and warrants further investigation. Assessing lactoferrin concentration in GCF holds marked potential as a diagnostic tool, offering insights into the presence of inflammation, the extent of oxidative stress and the effectiveness of periodontal treatment interventions (12). Lactoferrin exhibits a dual function in modulating immunological responses, functioning as a mediator with pro- or anti-inflammatory effects, contingent upon the situation and signalling pathways it engages (13). Due to this dual effect, the precise role of lactoferrin in periodontal disease remains unclear.

So far, to the best of our knowledge, one systematic review and meta-analysis has explored the role of lactoferrin in bone regeneration (14); however, there remains a lack of comprehensive reviews focusing on its potential as a diagnostic biomarker in periodontal disease. To address this gap, the present meta-analysis investigated the function of lactoferrin serving as an indicator of periodontitis by examining its association with key clinical indicators, including PPD, clinical attachment loss (CAL) and gingival index (GI). The present analysis aimed to clarify the diagnostic and prognostic value of lactoferrin in detecting disease presence and progression, thereby contributing to an improved understanding of its relevance in periodontal diagnostics. The clinical question in the present meta-analysis, formulated using the PICOS framework, aimed to determine whether lactoferrin levels vary between patients with periodontitis (Population) and healthy subjects (Comparison). The study assessed lactoferrin concentrations in saliva, serum or GCF (Outcome) with respect to the occurrence of periodontitis (Intervention), including only studies that used ELISA as the detection method (Study design). Furthermore, the analysis explored the association between lactoferrin levels, periodontal disease severity and standard clinical periodontal indicators to assess its potential role as a diagnostic and prognostic biomarker.

## Materials and methods

### Protocol development and research question

The methodology for the present systematic review and meta-analysis complied with Preferred Reporting Items for Systematic Reviews and Meta-Analyses (PRISMA) guidelines to ensure methodological rigor (15). The meta-analysis was registered with PROSPERO (https://www.crd.york.ac.uk/prospero/; registration no. CRD420251003457). The study design included case-control, cross-sectional, randomized controlled trial and non-randomized controlled trial studies that utilized ELISA as the detection method for lactoferrin. Only studies utilizing ELISA were included to ensure methodological consistency, enable robust quantitative comparisons and reduce heterogeneity stemming from assay-related variability. ELISA remains the most standardized, validated and widely adopted platform for lactoferrin detection in both clinical and translational research settings (16).

### Data collection and search procedures

A thorough and systematic electronic search was independently conducted by three researchers using major databases, including PubMed (https://pubmed.ncbi.nlm.nih.gov/), Google Scholar (https://scholar.google.com/), the Cochrane Library (https://www.cochranelibrary.com/) and ResearchGate (https://www.researchgate.net/); additional records were identified through manual screening of reference lists and citation tracking of relevant studies. The literature search was conducted between December 1, 2024 and February 28, 2025, ensuring thorough coverage of relevant literature. Additionally, pertinent studies were identified through hand-searching the bibliographies of the selected articles and related review articles. The following MeSH terms were used for searching: ‘Periodontitis’, ‘periodontal disease’, ‘lactoferrin’, ‘probing pocket depth’, ‘clinical attachment level’ and ‘gingival index’. Combining these terms with logical operators such as AND or OR search phrases such as [‘Periodontitis’ (MeSH) OR ‘Periodontal Diseases’(MeSH)] AND ‘Lactoferrin Protein’(MeSH)] [‘Periodontal Pocket’(MeSH) OR ‘Clinical Attachment Loss’(MeSH) OR ‘Gingival Index’(MeSH)] were used to identify relevant studies for the present meta-analysis.

### Eligibility criteria

Studies assessing the association between lactoferrin concentration and periodontal indicators such as PPD, CAL and GI in relation to periodontal disease were included. The studies included were original, peer-reviewed, full-text articles in English. Eligible studies involved human participants, used ELISA to quantify lactoferrin in biological fluids (serum, saliva or GCF), considered lactoferrin as a biomarker, and included periodontitis and healthy control groups for comparison. The present meta-analysis encompasses studies published between 1993 and 2022, offering a broad and longitudinal perspective on the evolving body of evidence. The present study excluded case reports, reviews, letters, commentaries and conference abstracts regardless of data availability, as well as unpublished, preprint or grey literature with poor methodology. In addition, animal or *in vitro* studies without human data were not considered. Research not focused on periodontal disease or without proper periodontal assessment was excluded, along with studies failing to measure lactoferrin levels, lacking a control group comparison, or not evaluating the diagnostic accuracy of lactoferrin. Non-English studies were also excluded unless a translation was available.

### Data extraction

**Figure 1:**
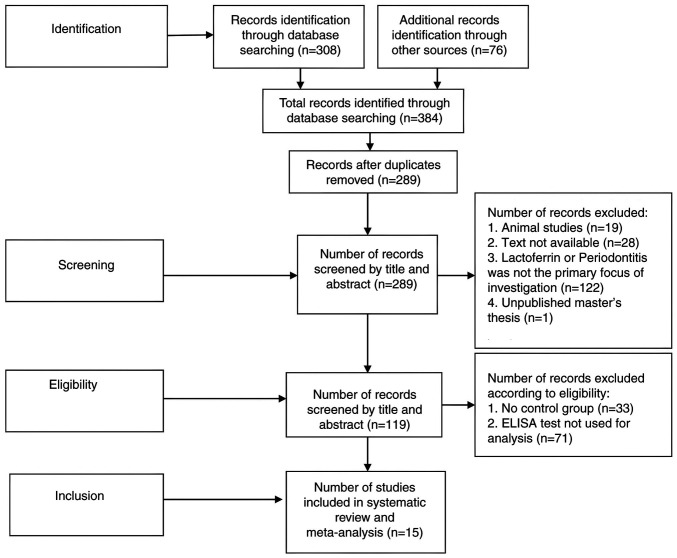
Preferred Reporting Items for Systematic Reviews and Meta-Analyses flowchart depicting the study selection process.

Independent, comprehensive searches of the literature were undertaken by the authors across various databases. The search process, outlined according to the PRISMA flowchart, is shown in Fig. 1.

### Evaluation of methodological quality

The robustness of the study methods and established tools were used to assess possible sources of bias: Case-control and cross-sectional studies were evaluated using the Newcastle-Ottawa Scale; randomized controlled trials were assessed with the Jadad Scale; and non-randomized controlled trials were appraised utilizing the Modified Methodological Index for Non-Randomized Studies (Modified MINORS) (17-19).

### Data items

Data from the included studies were exported into Microsoft Excel (Office 2013; Microsoft Corporation) for analysis. For the subsequent analysis, the authors independently extracted key information from each eligible study, which included: The first author, publication year, country, study design, sample type (serum, saliva or GCF), molecular analysis method (ELISA), measured lactoferrin levels, PPD, CAL and GI, and the total number of participants, including both controls and patients with periodontitis (Table I). Any discrepancies in study selection, quality assessment or data extraction were resolved through consensus discussions among the authors.

### Statistical analysis

The analysis included 15 studies, utilizing the odds ratio (OR) as the primary effect size index. The analysis employed a random-effects model, assuming that the included studies represent a random sample drawn from a broader population of relevant studies, which enables the results to be generalized beyond the specific data analysed (20). To evaluate the association between lactoferrin levels in saliva, serum or GCF and periodontitis, the standardized mean difference (SMD) with a 95% confidence interval (CI) was calculated, incorporating a random-effects model to account for potential heterogeneity among studies. For all outcomes, SMD and the corresponding CI were reported as the effect measures. Additionally, a prediction interval was computed to estimate the range within which the true effect in future studies might lie (21). The statistical significance of the pooled SMD between patients with periodontitis and healthy controls was assessed using a Z-test. Heterogeneity across the studies was assessed by applying the Q-statistic, I^2^-statistic, τ and τ^2^, along with visual examination of the forest plots (22). Publication bias was assessed both qualitatively and quantitatively through funnel plot analysis, Begg's rank correlation test, and Egger's linear regression test (23). Statistical analyses were executed with the aid of Comprehensive Meta-Analysis Software (version 4; https://meta-analysis.com). Two-tailed P<0.05 was considered to indicate a statistically significant difference.

## Results

### Overview of studies included. Number of studies

Overall, 384 studies were retrieved from various databases between December 1, 2024 and February 28, 2025. Among the 384 studies, 15 eligible studies, as determined by the inclusion and exclusion criteria, were included in the meta-analysis representing data from 798 participants (496 cases and 302 controls).

*Study characteristics*. The 15 included studies consisted of nine case-control studies, four cross-sectional studies, one non-randomized interventional study and one randomized controlled trial. Lactoferrin levels were evaluated across various studies in different biological fluids, including saliva, serum and GCF, to compare lactoferrin concentration and periodontal parameters (PPD, CAL and GI) between patients with periodontitis and healthy individuals. The studies were conducted across different regions of Asia, Europe and Africa, with publication dates ranging from 1993 to 2022. All studies assessed with the Newcastle-Ottawa Scale had quality scores of 7 or 8. For the randomized controlled trial and non-randomized interventional study, the Jadad scale and Modified MINORS scale were used as the Newcastle-Ottawa Scale cannot be used for these studies (17). The Jadad scale score was 4 for the randomized controlled trial and the Modified MINORS scale score was 13 for the non-randomized interventional study. Analysis revealed notable differences in lactoferrin levels and periodontal parameters comparing patients with periodontitis to healthy controls: Salivary lactoferrin was increased in 156 patients out of 230 in the test group; in GCF, lactoferrin levels were increased in 241 patients out of 297 patients in the test group; and serum lactoferrin was elevated in 15 patients compared with 15 controls. In addition, the clinical parameters (PPD, CAL, GI) were higher in 471 patients out of 496 patients in the test group.

### Overall effect size analysis

The pooled analysis of the 15 included studies revealed a significant association between periodontitis and elevated lactoferrin levels (SMD=2.630, P<0.010; 95% CI: 1.140-11.180) (Table II). Among these, 10 studies (9,11,12,24-30) reported statistically significant positive associations (P<0.05) with OR>1. The remaining five studies (31-35) did not show statistically significant associations. For PPD, the pooled SMD was 8.630 (P<0.010; 95% CI: 17.070-90.340) and 13 studies (11,12,24,25,27-35) demonstrated a statistically significant association between elevated lactoferrin levels and increased PPD in individuals with periodontitis (OR>1; P<0.05). In contrast, studies by Yadav *et al* (26) and Glimvall *et al* (9), despite reporting OR>1, did not show statistically significant associations, as their corresponding P-values were >0.05 (Table III). Similarly, for CAL, the pooled SMD was 8.382 (P<0.010; 95% CI: 19.855-123.053), with 14 studies (9,11,12,24,25,27-35) showing a significant association between elevated lactoferrin levels and increased CAL in individuals with periodontitis and one [Yadav *et al* (26)] reported no association (Table IV). Regarding GI, the pooled SMD was 4.288 (P<0.010; 95% CI: 2.914-17.654). Eight studies (9,11,12,24,27,29,30,35) demonstrated a statistically significant association between elevated lactoferrin levels and increased GI in individuals with periodontitis (P<0.05). Among the remaining studies, five reported (25,28,31,32,34) OR>1 but did not reach statistical significance (P>0.05). Shimizu *et al* (33) reported a neutral OR of 1 with P>0.05, whereas Yadav *et al* (26) showed an OR<1 with P>0.05, indicating no significant association (Table V). Table III, Table IV and Table V present the association between elevated lactoferrin levels and the periodontal clinical parameters PPD, CAL and GI in individuals with periodontitis.

### Heterogeneity

The heterogeneity analysis across the 15 included studies revealed substantial variability in outcomes under the random-effects model. For lactoferrin concentration, the Q-value was 153.664 (P<0.010) with 14 degrees of freedom and an I^2^ of 90.89%, indicating significant heterogeneity beyond chance. Similarly, PPD, CAL and GI showed Q-values of 103.060, 105.864 and 120.646, respectively (all P<0.010), with corresponding I^2^ values of 83.15, 85.83 and 88.40%, further supporting substantial heterogeneity across studies. Despite this, the between-study variance (τ^2^) remained low for all parameters (lactoferrin τ^2^=3.617; PPD τ^2^=2.060; CAL τ^2^=2.806; GI τ^2^=2.700), suggesting that the pooled effect sizes were relatively stable across different populations and study settings.

### Forest plots

**Figure 2:**
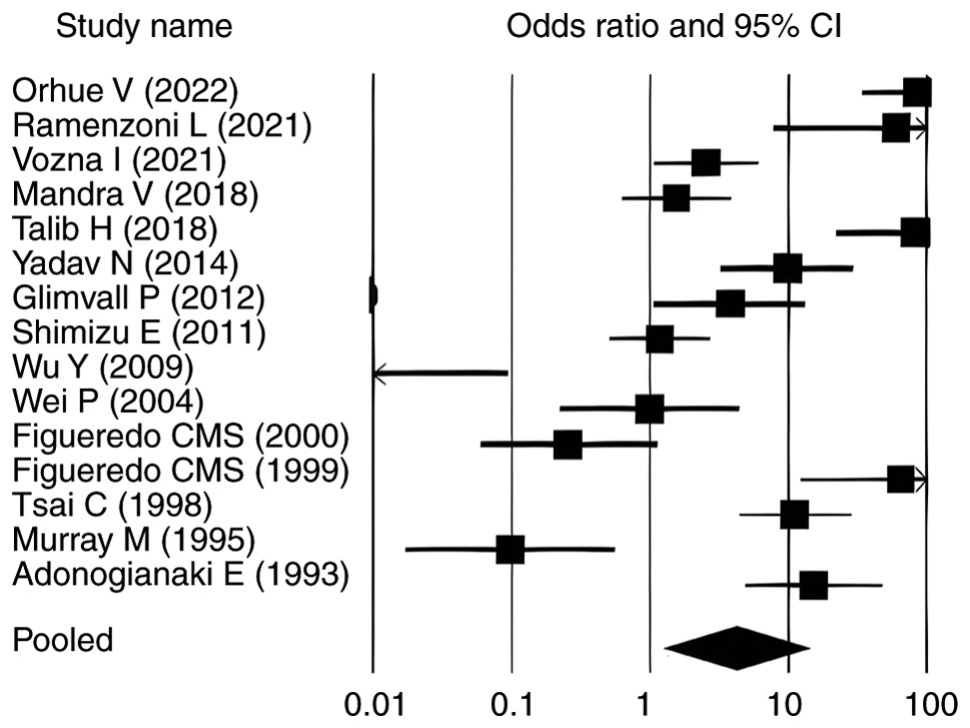
Forest plot illustrating the association of lactoferrin with periodontitis. Odds ratio values are shown as squares, and the horizontal lines represent the 95% CI for each study. CI, confidence interval.

**Figure 3:**
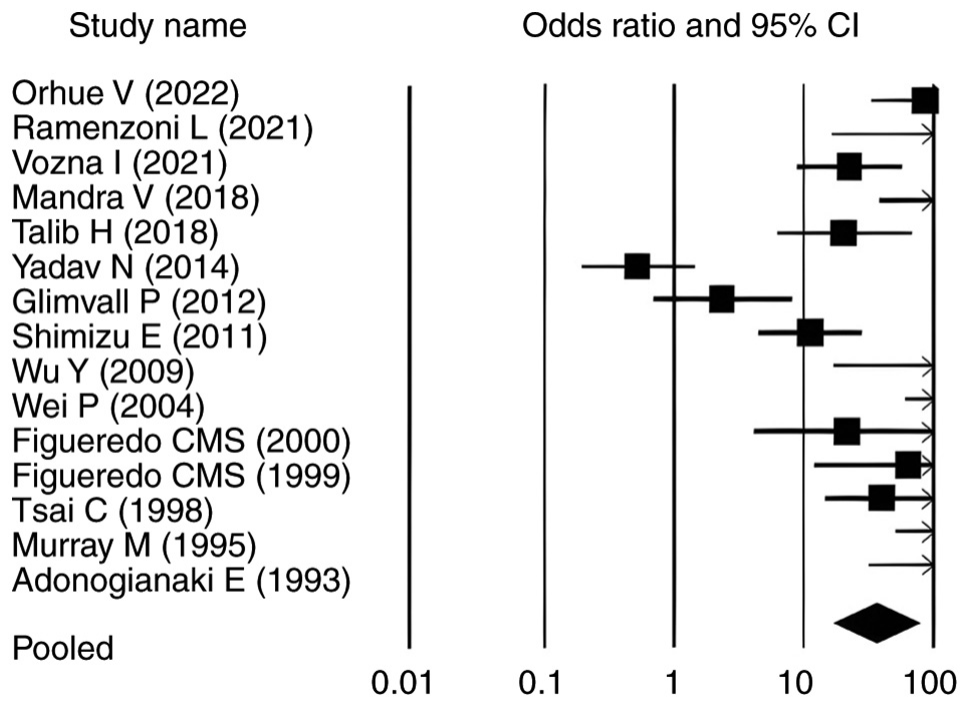
Forest plot depicting the association between lactoferrin concentrations and probing pocket depth as a clinical periodontal parameter. Odds ratio values are shown as squares, and the horizontal lines represent the 95% CI for each study. CI, confidence interval.

**Figure 4:**
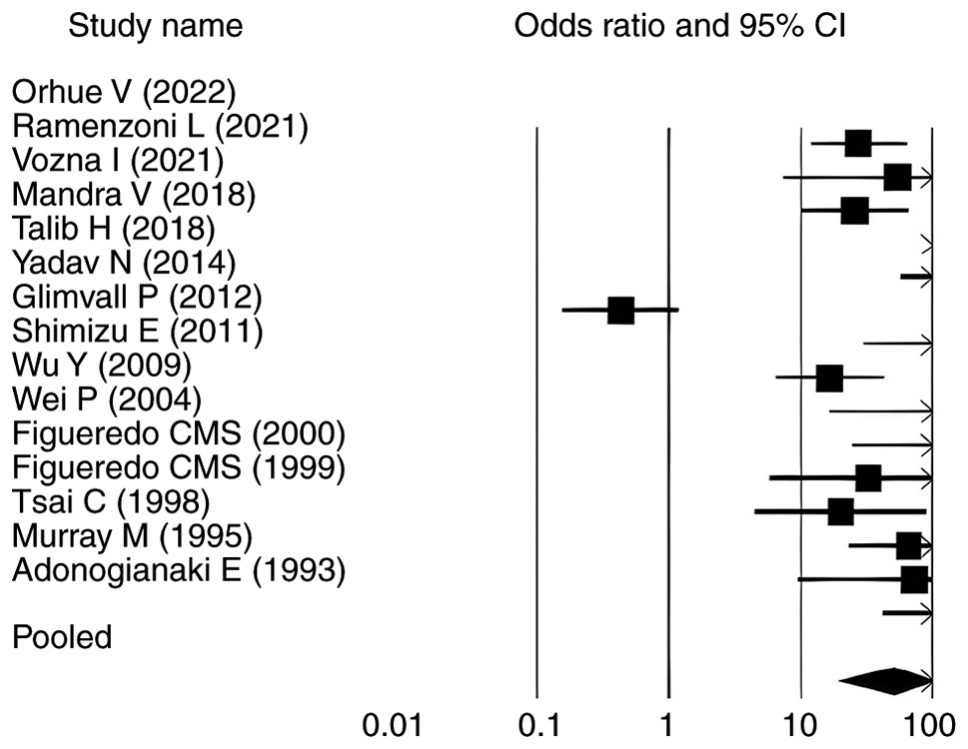
Forest plot showing the association between lactoferrin concentration and clinical attachment loss as a clinical periodontal parameter. Odds ratio values are shown as squares, and the horizontal lines represent the 95% CI for each study. CI, confidence interval.

**Figure 5:**
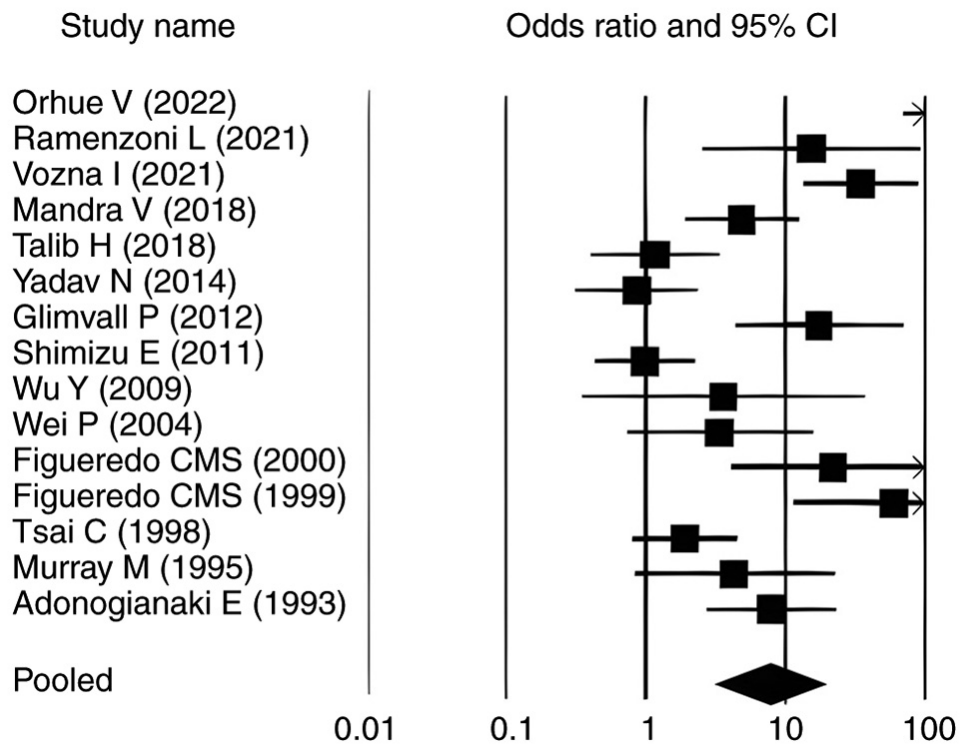
Forest plot depicting the association between lactoferrin concentration and gingival index as a clinical periodontal parameter. Odds ratio values are shown as squares, and the horizontal lines represent the 95% CI for each study. CI, confidence interval.

The forest plots for all analysed parameters, such as lactoferrin concentration, PPD, CAL and GI, illustrate individual study result, with the square indicating the OR and the line representing the 95% CI. In each plot, the pooled effect size is depicted by a diamond at the bottom, indicating a statistically significant positive association. In each plot, the pooled effect size is depicted by a diamond at the bottom, indicating a statistically significant positive association. For lactoferrin concentration, the CIs ranged from 1.140-11.180, whereas for PPD, CAL and GI in relation to elevated lactoferrin in individuals with periodontitis, the intervals were 17.070-90.340, 19.855-123.053 and 2.914-17.654, respectively (Fig. 2, Fig. 3, Fig. 4 and Fig. 5). Most individual study outcomes aligned closely with the pooled estimates, reflecting a generally consistent trend across studies. Although some studies displayed wider CIs, suggesting slight variance, the overall pattern supports a low level of heterogeneity and reinforces the robustness of the findings across different clinical indicators.

### Publication bias. Funnel plots

**Figure 6:**
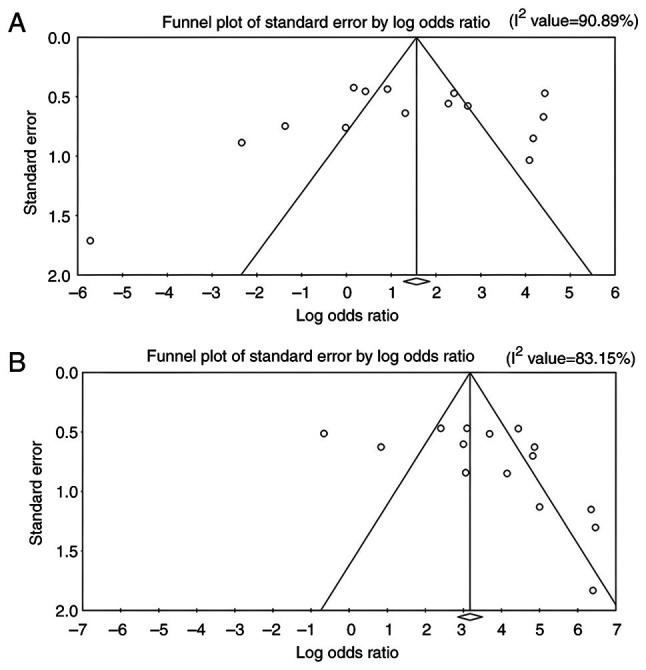
Funnel plots assessing potential publication bias for the analyzed outcome measures: (A) Lactoferrin concentration and (B) probing pocket depth.

**Figure 7:**
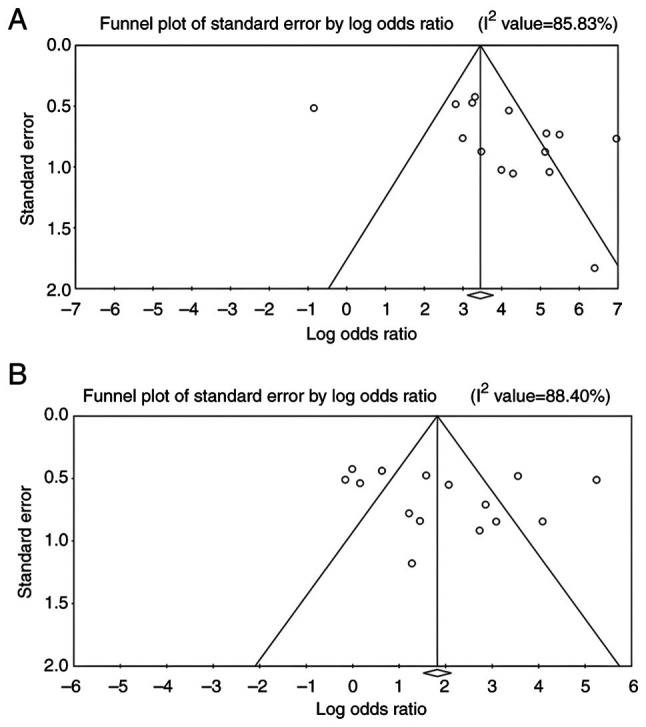
Funnel plots assessing potential publication bias for the analyzed outcome measures: (A) Clinical attachment level and (B) gingival index.

The funnel plots for all outcome measures, including lactoferrin concentration, PPD, CAL and GI, appeared visually symmetrical, indicating a balanced distribution of study effect sizes around the pooled estimates (Figs. 6 and 7). Although minor asymmetries were observed in a few studies, these did not suggest the presence of marked publication bias.

*Eggers's regression intercepts*. Potential publication bias across all outcome variables was assessed using Egger's test. No statistically significant evidence of publication bias was observed for lactoferrin concentration (T=-0.646; P=0.529). Likewise, Egger's test did not indicate evidence of small-study effects for PPD (T=0.335; P=0.743) or GI (T=0.930; P=0.370). By contrast, Egger's test for CAL yielded a statistically significant result (T=2.327; P=0.035), suggesting the possible presence of small-study effects, which may indicate publication bias or methodological heterogeneity among the included studies.

*Begg's rank correlation test:* The Begg's test results are interpreted solely in the context of publication bias. Specifically, for lactoferrin concentration, the Kendall's τ-value of -0.105 with P=0.620 indicated no evidence of publication bias. Similarly, no statistically significant evidence of publication bias was observed for PPD (τ=0.230; P=0.230), CAL (τ=0.510; P=0.610) or GI (τ=0.238; P=0.239). Overall, these findings suggest that publication bias is unlikely to have materially influenced the pooled estimates.

## Discussion

Periodontitis is a chronic inflammatory condition with multiple contributing factors, which is mainly triggered by the build-up of dental plaque, which progressively damages the periodontium (1). Lactoferrin serves a pivotal role in safeguarding oral and periodontal health by serving as a frontline defence protein, exerting antimicrobial effects, modulating immune responses and reducing inflammation (8). Lactoferrin influences cytokine activity by upregulating anti-inflammatory cytokines, including IL-4 and IL-10, while also modulating the production of pro-inflammatory cytokines such as TNF-α, IL-1, IL-6 and granulocyte-macrophage colony-stimulating factor (8,9). Lactoferrin levels have been shown to be associated with various periodontal indicators, such as the plaque index, GI, bleeding on probing, PPD and CAL. Elevated lactoferrin levels in saliva, serum and GCF have been recognized as potential biomarkers of periodontal disease activity, emphasizing its utility as both a diagnostic tool and a target for therapeutic intervention (12,24,27).

The present meta-analysis included 15 studies comprising saliva, serum and GCF samples from patients with periodontitis and healthy controls. The pooled analysis revealed significantly elevated lactoferrin levels in patients with periodontitis compared with controls, with a SMD of 2.630, demonstrating a strong positive association with disease presence. Elevated lactoferrin levels were associated with higher PPD, CAL and GI across the included studies. Across the 15 included studies, statistically significant findings (OR>1, P<0.05) were reported in 10 studies for lactoferrin concentration in individuals with periodontitis compared with healthy controls. Collectively, these findings suggest that elevated lactoferrin levels are a common feature of periodontitis, supporting its role as a marker of periodontal inflammation. For periodontal clinical parameters, statistically significant results were observed in 13 studies for PPD, 14 studies for CAL and 8 studies for GI. In the majority of included studies, OR>1 with statistically significant P-values were reported, indicating an overall positive association between elevated lactoferrin levels and worsening periodontal parameters in patients with periodontitis. A few studies, including Murray *et al* (32), Wu *et al* (31), Wei *et al* (34) and Figueredo and Gustafsson (35), reported neutral or slightly decreased lactoferrin levels, likely due to small sample sizes, dilution effects in whole saliva or proteolytic degradation by bacterial enzymes. Nonetheless, the majority of evidence consistently indicated elevated lactoferrin in periodontitis, underscoring its clinical relevance.

Studies by Legrand *et al* (36) and Glimvall *et al* (9) have demonstrated that elevated lactoferrin levels primarily derived from polymorphonuclear neutrophils are a consistent finding across various inflammatory conditions, including arthritis, inflammatory bowel disease and chronic periodontitis, reinforcing its relevance as a key biomarker. According to Actor *et al* (37), lactoferrin has a role in modulating adaptive immunity by attracting sentinel immune cells, driving the development of T-cell precursors into active T-helper cells and enhancing the maturation of immature B cells into competent antigen-presenting cells. Kivadasannavar *et al* (10) observed a notable decline in lactoferrin concentrations in GCF following periodontal surgery, suggesting that measuring lactoferrin levels in GCF could be an effective approach to monitor treatment progress in periodontitis.

The present meta-analysis specifically focused on the role of lactoferrin in periodontitis and its association with key periodontal indicators, including PPD, CAL and GI. By consolidating findings from the included studies, the meta-analysis indicates that elevated lactoferrin levels in saliva, serum and GCF are generally associated with increased periodontal disease severity, reflecting its antimicrobial activity through neutrophil recruitment, enhanced phagocytic bacterial clearance and modulation of immune responses during inflammation (30). These results underscore the potential of lactoferrin as a clinically relevant, non-invasive biomarker for early detection, risk stratification and monitoring of treatment response in periodontitis. Furthermore, its association with inflammatory activity highlights its utility in guiding therapeutic interventions and evaluating treatment efficacy, while future studies with standardized sampling, sensitive assays and longitudinal monitoring may support the development of lactoferrin-based diagnostic and prognostic platforms in routine clinical practice.

The present meta-analysis additionally revealed that elevated lactoferrin levels were consistently associated with increased periodontal indicators, such as PPD, CAL and GI in patients with periodontitis compared with healthy individuals. For periodontal clinical parameters, statistically significant results were observed in 13 studies for PPD, 14 studies for CAL and eight studies for GI. These studies documented OR>1 and statistically significant P-values (<0.05), reinforcing a robust association between increased PPD, CAL and GI, and the severity of periodontal disease. Additionally, potential variability in diagnostic criteria, sampling methods or unaccounted confounding factors may have weakened the observed association in pooled analyses. In support of the link between lactoferrin and disease severity, Orhue *et al* (12) demonstrated elevated salivary lactoferrin concentration in individuals with chronic periodontitis compared with healthy subjects, along with a positive association between lactoferrin and key periodontal parameters such as bleeding on probing and PPD.

Recent advances in bio-detection technologies can enhance the diagnostic potential of lactoferrin in periodontal disease. Nano-enabled platforms, including iron molybdenum disulfide-assisted lateral flow systems and nanozyme-based biosensors, offer improved sensitivity, speed and stability for detecting inflammatory biomarkers (38,39). Innovations such as ferric yolk-shell nanostructures and label-free metabolic profiling further increase diagnostic precision across biological samples (40). Together, these approaches enable rapid, non-invasive and high-resolution detection, supporting the development of lactoferrin-based assays for monitoring periodontal disease activity.

The present meta-analysis has several limitations: The number of studies examining the association between lactoferrin and periodontitis was limited, underscoring the need for further research. Analysis was restricted to saliva, serum and GCF, excluding dental plaque, which may provide additional insight. Substantial heterogeneity likely arose from differences in assay methods, sample collection, diagnostic criteria, study populations, immune responses and oral hygiene practices. In addition, although ELISA was widely used, variations in sensitivity and specificity may have affected results. In addition, Egger's test indicated the presence of small-study effects for CAL, suggesting that the pooled estimate may be influenced by publication bias or methodological heterogeneity among the included studies. Standardizing collection and assay protocols is essential, and future studies should clarify the biological role of lactoferrin and its potential as a biomarker for systemic inflammatory conditions.

In conclusion, the present findings highlight the notable involvement of lactoferrin in the development and progression of periodontitis, establishing it as a crucial modulator of inflammation with considerable therapeutic promise. The present study demonstrated a positive association between lactoferrin concentrations and periodontal parameters, including PPD, CAL and GI. Furthermore, the consistently elevated lactoferrin levels detected in saliva, blood serum and GCF highlight its potential as a non-invasive biomarker for diagnosing periodontitis.

## Figures and Tables

**Table I:** Study characteristics.

			Number of subjects		Concentration of lactoferrin, ng/ml		PPD, mm		CAL, mm		GI, %			
First author, year, country	Study type	Sample type	Case group	Control group	Case group	Control group	Case group	Control group	Case group	Control group	Case group	Control group	New-castle-Ottawa Scale	(Refs.)
Orhue, 2022, Nigeria	CS study	Saliva	51	51	6,740±590	5,270±610	5.1±0.7	2.1±0.60	4.2±0.06	1.4±0.9	1.8±0.58	0.6±0.08	8	(12)
Ramenzoni, 2021, Switzerland	CC study	Saliva	10	10	1,253±139.74	507.5±64	7.15±1.06	2.95±0.38	6.12±0.60	1.95±0.07	1.9±0.5	0.8±0.9	8	(11)
Vozna, 2021, Ukraine	CC study	GCF	126	20	303.19±75.31	16.87±30.34	5.2±0.8	1.9±0.4	4.3±0.04	1.2±0.07	1.6±0.6	0.5±0.08	7	(24)
Mandra, 2018, Russia	CC study	Saliva	45	30	6,188.2±928.3	756.53±100.6	6.2±0.9	2.2±0.52	4.1±0.05	1.99±0.09	1.5±0.05	0.8±0.07	7	(30)
Talib, 2018, Iraq	CC study	Saliva	33	15	47.35±2.4	3.9±0.64	0.93±0.5	0.1±0.50	0.91±0.07	0.2±0.08	0.95±0.8	0.89±0.6	8	(25)
Yadav, 2014, India	NRI study	GCF	25	25	1,857.21±91.5	75.34±7.13	0.55±0.7	0.825±0.8	0.50±0.7	0.73±0.07	0.67±0.02	0.72±0.9	13^a^	(26)
Glimvall, 2012, Sweden	CC study	Saliva	17	17	2,710±2,360	1,120±1,980	0.45±0.6	0.25±0.08	0.41±0.08	0.21±0.06	0.9±0.04	0.8±0.08	8	(9)
Shimizu, 2011, Japan	RCT	Saliva	37	35	9,800±6500	9,300±3,500	4.16±0.09	4.04±0.09	4.18±0.09	4.04±0.09	1.34±0.06	0.34±0.06	4^b^	(33)
Wu, 2009, China	CC study	Saliva	5	5	6,800±800	8,900±500	5.6±1.02	2.2±0.9	5.2±0.4	1.6±0.05	1.4±0.6	0.9±0.8	7	(31)
Wei, 2004, Taiwan	CC study	GCF	19	8	192,750±156,540	193,250±168,120	5.12±1.04	1.86±0.55	4.9±0.6	1.08±0.07	2.48±0.55	0.1±0.6	7	(34)
Figueredo, 2000, Sweden	CC study	GCF	13	12	237.1±110.9	352.8±191.1	5.5±0.6	2.4±0.4	4.2±0.7	1.9±0.08	1.9±0.3	1.3±0.4	7	(35)
Figueredo, 1999, Sweden	CC study	Serum	15	15	558±96	193±32	5.3±1.03	2.6±0.2	4.1±0.6	1.2±0.08	1.8±0.5	0.9±0.2	8	(27)
Tsai, 1998, Taiwan	CS study	GCF	66	23	552.16±419.4	69.36±22.2	5.69±1.8	2.39±0.50	5.2±0.6	1.9±0.06	2.40±0.61	0.22±0.42	8	(28)
Murray, 1995, UK	CS study	GCF	10	10	106.2±193.9	619.92±531.7	5.8±1.81	0.9±0.69	5.3±0.4	1.8±0.8	2.3±0.82	0.1±0.31	7	(32)
Adonogianaki, 1993, UK	CS study	GCF	24	26	217±111	95±37	5.04±0.29	1.54±0.10	4.9±0.5	1.1±0.5	2.46±0.17	0.38±0.10	7	(29)

^a^Modified Methodological Index for Non-Randomized Studies score;

^b^Jadad score. CS, cross-sectional; CC, case-control; RCT, randomized controlled trial; NRI, non-randomized interventional; GCF, gingival crevicular fluid; PPD, pocket depth; CAL, clinical attachment loss; GI, gingival index.

**Table II:** Pooled ORs with 95% CIs, Z-values and P-values for lactoferrin levels across included studies under a random-effects model.

First author, year	OR	95% CI	Z-value	P-value	(Refs.)
Orhue, 2022	85.046	33.511-215.835	9.351	<0.010	(12)
Ramenzoni, 2021	60.171	7.866460.274	3.351	<0.010	(11)
Vozna, 2021	2.528	1.067-5.988	2.108	<0.010	(24)
Mandra, 2018	1.544	0.628-3.799	0.946	<0.010	(30)
Talib, 2018	82.474	21.996-309.231	6.544	<0.010	(25)
Yadav, 2014	9.857	3.278-29.642	4.073	<0.010	(26)
Glimvall, 2012	3.758	1.067-13.240	2.061	0.039	(9)
Shimizu, 2011	1.188	0.514-2.749	0.403	0.687	(33)
Wu, 2009	0.003	0.000-0.096	-3.327	0.001	(31)
Wei, 2004	0.994	0.222-4.449	-0.007	0.994	(34)
Figueredo, 2000	0.257	0.059-1.121	-1.808	0.710	(35)
Figueredo, 1999	65.531	12.277-349.797	4.895	<0.010	(27)
Tsai, 1998	11.184	4.408-28.375	5.083	<0.010	(28)
Murray, 1995	0.097	0.017-0.559	-2.614	0.009	(32)
Adonogianaki, 1993	15.186	4.862-47.426	4.682	<0.010	(29)
Random-effects model	3.960	1.140-11.180	2.630	<0.010	

OR, odds ratio, CI, confidence interval.

**Table III:** Pooled ORs with 95% CIs, Z-values and P-values for probing pocket depth across included studies under a random-effects model.

First author, year	OR	95% CI	Z-value	P-value	(Refs.)
Orhue, 2022	85.763	33.768-217.818	9.361	<0.010	(12)
Ramenzoni, 2021	150.129	16.265-1385.759	4.419	<0.010	(11)
Vozna, 2021	22.311	8.831-56.368	6.566	<0.010	(24)
Mandra, 2018	331.138	80.674 451.545	7.729	<0.010	(30)
Talib, 2018	20.306	6.184-66.676	4.963	<0.010	(25)
Yadav, 2014	3.174	0.823-8.437	-1.426	0.154	(26)
Glimvall, 2012	2.334	0.678-8.032	-1.340	0.179	(9)
Shimizu, 2011	11.224	4.026 31.307	4.330	<0.010	(33)
Wu, 2009	608.723	16.655-222,482.563	3.492	<0.010	(31)
Wei, 2004	61.250	6.106-558.127	3.369	<0.010	(34)
Figueredo, 2000	21.557	4.106-113.174	3.629	<0.010	(35)
Figueredo, 1999	49.358	11.337-215.196	4.876	<0.010	(27)
Tsai, 1998	40.351	14.583-111.617	7.120	<0.010	(28)
Murray, 1995	649.82	50.210-8410.164	6.853	<0.010	(32)
Adonogianaki, 1993	125.443	31.500-499.535	4.988	<0.010	(29)
Random-effects model	39.270	17.070-90.340	8.630	<0.010	

OR, odds ratio; CI, confidence interval.

**Table IV:** Pooled ORs with 95% CIs, Z-values and P-values for clinical attachment loss across included studies under a random-effects model.

First author, year	OR	95% CI	Z-value	P-value	(Refs.)
Orhue, 2022	27.661	11.959-63.979	7.760	<0.010	(12)
Ramenzoni, 2021	54.683	7.281-410.686	3.890	<0.010	(11)
Vozna, 2021	25.851	10.164-65.746	6.829	<0.010	(24)
Mandra, 2018	1,064.485	23.739-4,827.187	9.037	<0.010	(30)
Talib, 2018	244.912	57.657-1,040.328	7.454	<0.010	(25)
Yadav, 2014	0.432	0.156-1.197	-1.613	0.107	(26)
Glimvall, 2012	169.051	30.138-948.250	5.831	<0.010	(9)
Shimizu, 2011	16.801	6.455-43.730	5.781	<0.010	(9)
Wu, 2009	607.663	16.636-2,2196.580	3.491	<0.010	(31)
Wei, 2004	189.269	24.377-1,469.517	5.014	<0.010	(34)
Figueredo, 2000	32.631	5.843-182.234	3.971	<0.010	(35)
Figueredo, 1999	20.176	4.482-90.816	3.915	<0.010	(27)
Tsai, 1998	66.449	23.050-191.561	7.768	<0.010	(28)
Murray, 1995	73.833	9.272-587.925	4.064	<0.010	(32)
Adonogianaki, 1993	174.835	41.956-728.564	7.091	<0.010	(29)
Random-effects model	49.430	19.855-123.053	8.382	<0.010	

OR, odds ratio; CI, confidence interval.

**Table V:** Pooled ORs with 95% CIs, Z-values and P-values for gingival index across included studies under a random-effects model.

First author, year	OR	95% CI	Z-value	P-value	(Refs.)
Orhue, 2022	191.968	70.058-526.017	10.222	<0.010	(12)
Ramenzoni, 2021	15.496	2.555-93.978	2.980	0.003	(11)
Vozna, 2021	35.314	13.680-91.159	7.366	<0.010	(24)
Mandra, 2018	4.91	1.920-12.555	3.322	0.001	(30)
Talib, 2018	1.183	0.410-3.415	0.310	0.756	(25)
Yadav, 2014	0.861	0.315-2.355	-0.291	0.771	(26)
Glimvall, 2012	17.599	4.353-71.151	4.024	<0.010	(9)
Shimizu, 2011	1.000	0.432-2.312	0.000	>0.999	(33)
Wu, 2009	3.606	0.355-36.602	1.085	0.278	(31)
Wei, 2004	3.391	0.7832-15.711	1.561	0.119	(34)
Figueredo, 2000	22.132	4.200-116.632	3.652	<0.010	(35)
Figueredo, 1999	60.125	11.419-316.585	4.833	<0.010	(27)
Tsai, 1998	1.894	0.797-4.502	1.446	0.148	(28)
Murray, 1995	4.293	0.822-22.416	1.728	0.084	(32)
Adonogianaki, 1993	7.978	2.695-23.623	3.750	<0.010	(29)
Random-effects model	7.173	2.914-17.654	4.288	<0.010	

OR, odds ratio; CI, confidence interval.

## Data Availability

The data generated in the present study may be requested from the corresponding author.
